# Combined Methods for Diabetic Retinopathy Screening, Using Retina Photographs and Tear Fluid Proteomics Biomarkers

**DOI:** 10.1155/2015/623619

**Published:** 2015-06-29

**Authors:** Zsolt Torok, Tunde Peto, Eva Csosz, Edit Tukacs, Agnes M. Molnar, Andras Berta, Jozsef Tozser, Andras Hajdu, Valeria Nagy, Balint Domokos, Adrienne Csutak

**Affiliations:** ^1^Department of Computer Graphics and Image Processing, Bioinformatics Research Group, Faculty of Informatics, University of Debrecen, Egyetem tér 1, Debrecen 4032, Hungary; ^2^Department of Ophthalmology, Faculty of Medicine, University of Debrecen, Egyetem tér 1, Debrecen 4032, Hungary; ^3^Astridbio Technologies Inc., 439 University Avenue, Toronto, ON, Canada M5G 1Y8; ^4^NIHR Biomedical Research Centre, Moorfields Eye Hospital NHS Foundation Trust and UCL Institute of Ophthalmology, 162 City Road, London EC1V 2PD, UK; ^5^Department of Biochemistry and Molecular Biology, Proteomics Core Facility, Faculty of Medicine, University of Debrecen, Egyetem tér 1, Debrecen 4032, Hungary; ^6^Centre for Research on Inner City Health, Keenan Research Centre, Li Ka Shing Knowledge Institute, St Michael's Hospital, 30 Bond Street, Toronto, ON, Canada M5B 1W8; ^7^InnoTears Ltd., Szent Anna Utca 37/1. 2. em. 1, Debrecen 4024, Hungary

## Abstract

*Background*. It is estimated that 347 million people suffer from diabetes mellitus (DM), and almost 5 million are blind due to diabetic retinopathy (DR). The progression of DR can be slowed down with early diagnosis and treatment. Therefore our aim was to develop a novel automated method for DR screening.* Methods*. 52 patients with diabetes mellitus were enrolled into the project. Of all patients, 39 had signs of DR. Digital retina images and tear fluid samples were taken from each eye. The results from the tear fluid proteomics analysis and from digital microaneurysm (MA) detection on fundus images were used as the input of a machine learning system.* Results*. MA detection method alone resulted in 0.84 sensitivity and 0.81 specificity. Using the proteomics data for analysis 0.87 sensitivity and 0.68 specificity values were achieved. The combined data analysis integrated the features of the proteomics data along with the number of detected MAs in the associated image and achieved sensitivity/specificity values of 0.93/0.78.* Conclusions*. As the two different types of data represent independent and complementary information on the outcome, the combined model resulted in a reliable screening method that is comparable to the requirements of DR screening programs applied in clinical routine.

## 1. Introduction

It is estimated that today more than 347 million people suffer from diabetes mellitus (DM) globally [1]. Diabetic retinopathy (DR), one of the most common complications of DM, accounts for about 5% of world blindness; this represents almost 5 million people in 2002. Approximately 80% of all patients with DM duration of at least 10 years suffer from some degree of DR [2]. During the development of DR, patients may not notice changes of their vision and DR might be very advanced by the time patients have visual complaints and experience visual loss eventually.

In order to detect DR in an early stage everyone with DM should be subjected to comprehensive dilated eye exam at least once a year [3]. In case of early diagnosis, the progression of DR can be slowed down by appropriate systemic or local therapy.

More than two-thirds of patients with DM live in developing countries such as India (32 million) and China (21 million) where the access to high quality health care system is not universal [4]. As a result of the high human resource cost of the DR screening programs, developed countries are also looking for more cost-effective and scalable alternatives to alter the existing methods.

Involvement of human graders is currently universal and the less automation is in the system the greater the costs are. Where possible, the digital photography method is the screening method of choice [5], although many countries rely on traditional clinical examination.

Recent photographic methods employ digital images evaluated by qualified personnel. Based on mydriatic 45° retinal photographs examined by trained staff, the sensitivity for the detection of sight-threatening DR has been reported to range between 87 and 100% with specificities of 83–96% [6]. The expected values for DR screening program as specified by the British Diabetic Association (Diabetes UK) are at least 80% sensitivity and 95% specificity [7, 8].

Quality control concerns and cost efficiency have brought forward the need for regular and centralized DR screening in several developed countries [9]. Digital images are captured at healthcare facilities and evaluated at grading centers by qualified personnel or ophthalmologists [10]. The process performs with high accuracy; however, it may not be scalable and sustainable in economically challenged countries as it requires intensive technical competence [11].

To improve the cost-effectiveness of DR screening several image processing based methods have been developed in the last decade providing an alternative to first phase examinations performed by human graders [12, 13]. In this case, the role of human graders would be reduced to examine true positive or ambiguous images as well as perform quality control on images that were described as normal by the software after the automated prescreening process [14].

The hallmark of DR is microaneurysms (MAs). As these outpouches occur on small blood vessels, most of the image processing based screening methods concentrate on the detection of this type of lesion. The International Retinopathy Online Challenge offers an opportunity to compare the results of image processing based algorithms for DR identification via MA detection, where a screening system developed by our research group achieved the best outcome in 2010 [15]. The specificity and sensitivity values of the automated MA detectors are close to those of human graders [16–18]. Despite the promising initial results, the use of image processing based methods is limited in the clinical routine, probably because their sensitivity and specificity cannot be improved further. Nevertheless automated image clarity assessment has been used in some places prior to human grading, for example, the Scottish Screening Programme [19].

In this study, in order to increase the sensitivity and specificity values of the photographic screening method, we used an MA detector combined with tear fluid proteomics based methodologies in one single system. The rationale behind this was to use two independent but complimentary techniques.

Currently tear fluid proteomics based methods are not used in the clinical routine either. Protein profile changes in tear fluid under abnormal pathological and physiological conditions, such as inflammatory diseases or wound healing, have been verified by several studies [20].

While vitreous humour proteome changes are known to be more specific [21], implementing vitreous humour proteomics in daily clinical practice is nevertheless problematic as it requires invasive method of sampling.

However, tear fluid sampling may be an efficiently standardizable noninvasive process. In our previous study, we analyzed the alteration of tear protein concentrations in DR in order to determine which proteins, as potential biomarkers, are found in tear fluid in DR patients [22].

In a previous paper our research group published the first attempt for using tear fluid proteomics multimarkers for DR screening. We applied machine learning algorithms to predict whether the given patients with DM suffer from DR or not, based on the global proteomics pattern changes of their tear fluid. In that study we concluded that, due to its low sensitivity (74%) and specificity (48%) values, the proteomics based screening method alone is not appropriate for clinical application at its present format. However, in combination with other methods, it is able to improve the performance of a combined system [23].

## 2. Materials and Methods

In the study we developed three automated screening systems. The first used image processing based algorithms in order to detect MAs in digital retina images. The second was based on the evaluation of tear fluid protein multimarkers. The third system combined the above mentioned MA detection and tear fluid proteomics analysis. Thereafter we assessed the performance of all three methods. Our aim was to develop an automated DR screening system that reaches the sensitivity and specificity values of screening performed by human graders. In this way, compared to our previous work, this new method represents significant improvement.

### 2.1. Patient Examination

Altogether, 52 patients with DM were recruited into the study (21 males; 65.2 years average age; 16.4 years average duration of diabetes; 14 NIDDM) from the Ophthalmology Outpatient Clinic of the University of Debrecen. At the time of the patient examination all the patients were under antidiabetic medication. Out of all patients, 39 had signs of DR. 16 patients out of the 39 have undergone one or more laser photocoagulation treatment. In our study, we only involved the eyes of the patients of which we could obtain complete tear fluid proteomics data and clinically evaluable fundus photos. Out of the potential 104 eyes, 74 had corresponding tear fluid analysis and gradable digital retinal images. Although the remaining 30 eyes were also examined clinically, either difficulties in tear fluid sampling, for example, noncompliance, operated eye, and keratoconjunctivitis (9 participants), or difficulties during the digital retina photography, for example, hemorrhage, angle-closure glaucoma, and cataract (21 participants) led to no procurement of relevant corresponding information as shown in Table 1.

DR was determined by first capturing and then grading standard 7-field fundus images. The images were taken by Megaplus Camera Model 1,6i/10 BIT Zeiss (Carl Zeiss Ophthalmic System A6, Jena, Germany). All of these were assessed by two independent ophthalmologists.

The collected tear fluid samples were obtained under standardized conditions by a qualified assistant [24]. The samples were acquired using glass capillaries directly before the pupil dilatation from the lower tear meniscus (a horizontal thickening of the precorneal tear film by the lower margin) at the lateral canthus and care was taken not to touch the conjunctiva. The time of the sampling procedure was noted. The secretion rate was calculated by dividing the collected tear volume by the time of sample collection and was given in *μ*L/min. Samples used in this analysis had secretion rates of 5–15 *μ*L/min.

### 2.2. Machine Learning

During the project data processing was performed by using machine learning algorithms. Machine learning is a subfield of artificial intelligence and it deals with the question as to how to construct computer programs that can learn from data. The core task of machine learning is performing inference from new samples. In supervised learning the goal is to predict the value of an outcome measure based on a number of input measures. Supervised learning takes a known set of input data (training set) and known responses (labeled output) to the data and seeks to build a predictor model that generates reasonable predictions for the response to new data.

In our case, the input data come from proteomics experiments and from image processing based method, while the predicted outcomes concern whether the certain patient has DR or not. The model makes predictions using new data to classify patients according to their tear proteomics data and digital retinal image gradings and forms a well-defined way to allow prescreening activities.

In the scientific literature no report can be found about the possibility and potential relevance of combining tear fluid proteomics and image processing on DR screening; the method proposed by this paper is unprecedented.

### 2.3. Microaneurysm Detector

**Figure 1:**
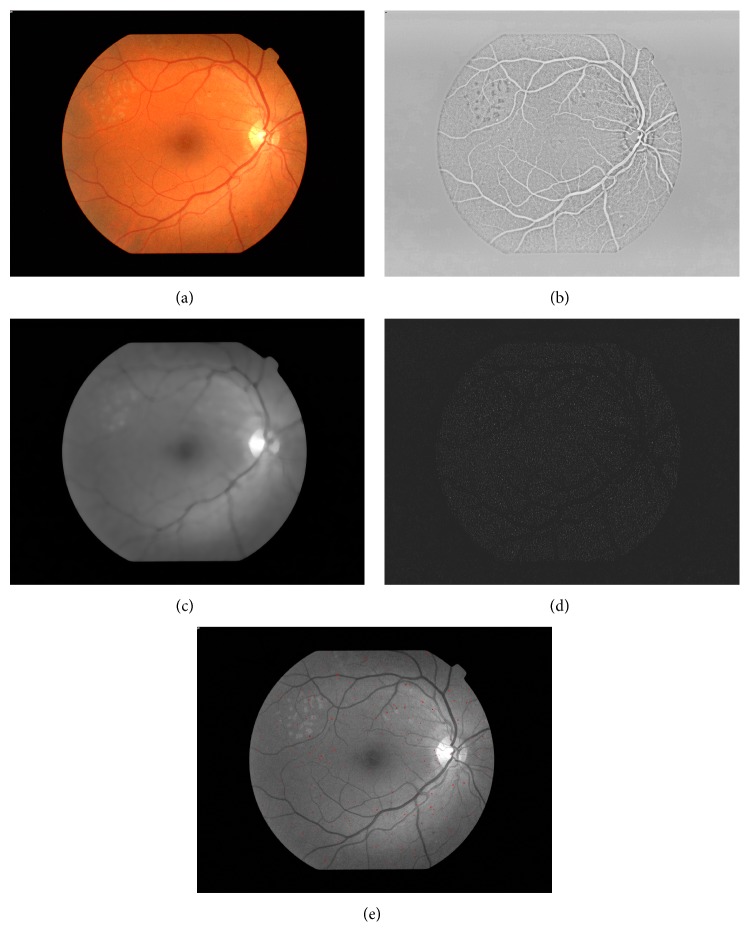
Microaneurysm detection. (a) Original retina image; (b) CLAHE contrast enhancement; (c) median filtering; (d) top-hat transform; (e) raw MA candidates.

The image data was processed using an MA detector, based on the image processing techniques (Figure 1) [25, 26]. In short, the green plane of the image is shade corrected, by subtracting the median filtered version of the image (using a 35 × 35 rectangle) from the green component of each pixel value. On the shade corrected image, a contrast limited histogram equalization (CLAHE) is performed, which is used to enhance the contrast of the image.

This was followed by a 3 × 3 median filtering for smoothing. The next step of the processing is a top hat transform by morphological reconstruction [27]. Top hat transform is an image processing method used for small feature extraction.

The reconstructed image is opened by a 10 × 10 disk shape to detect only small circular objects, resulting in candidate MAs. The center of the candidates is calculated and the following features are extracted for each candidate: area, rotational inertia, mean intensity in the morphologically opened image, mean intensity in green plane, and standard deviation of intensity in green plane.

The Gradient Boosting Machine (GBM) model was used to classify candidates MAs based on the above features [28, 29].

The testing was done based on hand marked MAs of the retina images.

### 2.4. Tear Fluid Proteomics Based Method

Immediately after sampling, the samples were centrifuged (1800 rpm) for 8–10 minutes and supernatants were deep-frozen at −80°C and were thawed only once for measurements.

Protein identification was done from the tear fluid of each of the patients. Tear samples were examined using nano-HPLC coupled ESI-MS/MS mass spectrometry protein identification as described elsewhere [22, 30, 31].

### 2.5. Application of Machine Learning Methods

Machine learning method provided the basis of our DR screening procedure. We have used learning data sets in order to teach the machine learning algorithms to predict the occurrence of certain future events, using empirical data containing incomplete information. In our case the learning datasets were the proteomics profiles, the retinal images, and the clinical diagnosis (DR/non DR) of the enrolled diabetes patients with DM.

Three classifiers were trained on the datasets; the first model was based on fundus image data alone, the second on using proteomics data, and the final was the combined model. During the process we used the best performing machine learning algorithms, Naive Bayes Classifier in the first and GBM in the other two models. 10-fold cross-validation (repeated 10 times) was used to assess the performance of this classifier.

The fundus images were processed using the MA detector algorithm, and a threshold was chosen for a candidate to be classified as MA. The count of MAs on an image found this way was used as the only feature for detecting DR, in case of the first model. In Table 2, our results are reported at image level.

We have used naive Bayes classifier for this analysis.

In the second model the global pattern of protein concentrations and its changes were described by the examination of the concentrations of 34 different proteins.

For the combined data analysis our input data were protein levels measured in tear fluid samples from patients with diabetes, the number of detected MAs in the associated images, and clinical data regarding their DR status.

**Figure 2:**
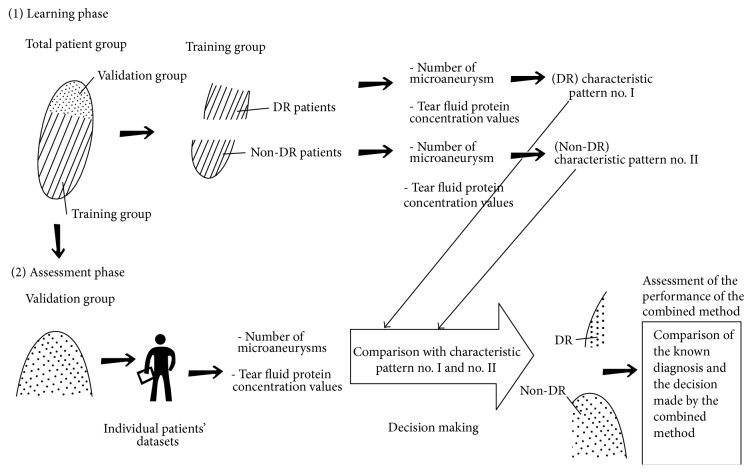
Application of machine learning algorithm in the combined model. Learning phase (above): we randomly select a subpopulation of the total patient group, called the training group, and then use the known clinical diagnosis to split the training group into a DR group and a non-DR group. The clinical diagnosis, the number of MAs on the retina images, and the protein concentration values are the inputs of the machine learning algorithm. The algorithms are able to tell which data patterns are the most characteristic for the DR and non-DR groups. Assessment phase (below): in the following steps, we use the data from the validation group. The number of MAs and the protein concentration values constitute the input of the algorithm, but we do not use the information from clinical diagnosis. The learning algorithm compares the new data to the characteristic patterns that are known from the learning phase and will make its own decision (normal/DR) for each patient as the output of the model. For the assessment of the performance of the model, we compare the output with the known clinical diagnosis.

As a next step, following the learning process, we intended to assess the performance of the screening method. At this phase only the protein levels and retinal images were entered into the system without diagnosis. Our goal was to show that the model based on both types of data has better performance than the models based on either the image data or the proteomics data alone.  Figure 2 shows the learning and the assessment phases of the application of the machine learning algorithm.

We used* k*-fold cross-validation method to evaluate the classifier's performance based on the three dataset setup. In* k*-fold cross-validation the data is first partitioned into* k* equally (or nearly equally) sized segments or folds [32].

Afterwards* k* iterations of training and validation are performed. Within each iteration different subsets of the data are kept for validation process while the remaining *k* − 1-fold are used for learning. At the end of the cross-validation, the estimate is determined as the mean of the features of the* k*-fold model where we used 10-fold cross-validation, repeated 10 times.

With this basic form of cross-validation we obtained estimations for many performance indicators such as specificity, sensitivity, accuracy, and* F*-measure (harmonic mean of precision and sensitivity as a single measure for the performance of the model).

During the 10-fold cross-validation process, the content of learning and validation datasets has been randomly chosen. In certain cases, one eye of a person was assigned to the validation and the other to the learning dataset. Considering the relatively low number of participants enrolled in the project, this setting may increase significantly the performance of the image processing based method. Therefore, we tried to exclude this potential bias from our experiments. When the software splits the total patient group into a validation and training set, we exclude the eyes from the training set whose pair was in the validation set.

### 2.6. Data Analysis: Software Tools

We used the Smartbiobank data management system for the collection of experimental (proteomics) and clinical data (DR/Non-DR) with the related retinal images [33]. During the data analysis process the *R* statistical framework and the following packages have been used: “gbm”, “caret”, “stat”, and “e1071” [34]. The image processing tasks were done using the Octave software [35].

## 3. Results and Discussion

In case of the first model MA-count was used as the only feature for detecting DR. MA detection method alone resulted in 0.84 sensitivity and 0.81 specificity values.

Using the proteomics data for analysis 0.87 sensitivity and 0.68 specificity values were achieved by using GBM models.

The combined model resulted in a more powerful classifier, achieving 0.93 sensitivity and 0.78 specificity values, as the two different types of data provide independent and complementary aspects of the underlying information of the outcome.

Data in Table 2 demonstrate that the MA detector alone performs significantly better than the tear proteomics based method. Regarding accuracy measurement, the combined method exceeds both image processing and proteomics based methods values. Specificity value of the MA detector slightly outperforms the specificity of the combined method as well. Nevertheless, taking everything into consideration, the use of proteomics data definitely improves the performance of the MA detector; therefore the combined screening method is the best classifier out of the three.

Over the last decade, several studies have been published on the application of image processing methods for DR screening. These methods, despite their promising performances, are only slowly being applied in clinical practice.

In parallel, our team published the first attempt of using tear fluid proteomics based method for DR screening. Although the conclusion of this work was that this method alone is not accurate enough for clinical use [23], there is a definite scope for the improvement of the performance of proteomics based classifier. With improvements in tear fluid analysis, this method might become clinically useful with time, as it requires little equipment for obtaining the sample making potential mass-screening of DR in hard-to-reach areas viable.

In theory, the performance of a classifier might be improved by using the combination of different predictor models [36, 37]. Combined diagnostic approaches are also used in the routine medical practice to improve the clinical effectiveness of existing screening programs [38].

Although there are numerous screening methods published to be comparable to human graders, there is a lack of systems that have been validated on independent cohort using internationally recognized DR standard. Abramoff and his workgroup published a method using combined MA, hemorrhage, cotton wool spots, and exudates detection on a diabetes at risk cohort of 874 participants. They completed the method with the detection of irregular lesions such as large hemorrhages and neovascularization. The sensitivity of the system was 96.8% (95% CI, 94.4%–99.3%) with 59.4% (95% CI, 55.7%–63.0%) specificity. The relatively low sensitivity value limits the usability of the system [39].

The automated grading method published by Goatman and his colleagues intended to remove normal images from the image database before manual grading, thus reducing the manual workload. The combined method was based on the detection of MA, blot hemorrhages, and exudates. According to the well-known tradeoff between sensitivity and specificity, the 100% sensitivity is coupled with low specificity value.

Considering that the system is proposed to be a prescreening tool, the workload reduction, ranged from 26.4% (MA/BH/EX, both fields) to 38.1% (MA only, macular field), is remarkable [40].

As we mentioned earlier, tear fluid proteomics based methods are not used in the clinical routine. In experimental settings the tear fluid proteomics based screening methods, because of the expensive MS/MS experiments, limit the application of larger sample sizes that are used in image processing based projects.

Protein biomarkers and the MAs on retinal images represent different data sources and information on DR eye. Our results of 0.93 sensitivity and 0.78 specificity values are close to reach the threshold recommended for routine clinical screening of 80% and 95%, respectively. Considering the 9 eyes for tear fluid proteomics examination and the 21 eyes for which the image processing cannot be performed, the clinical examination protocol should be improved in the future. Our future aim is to decrease the number of the protein biomarkers applied in the screening system in order to replace the MS/MS method to a more cost-effective rapid test. To keep the performance of the method we use Principal Component Analysis (PCA) to select the potential biomarkers with the highest predictive values. For this lower number of protein marker we intend to define a concentration threshold instead of using the absolute values of the concentrations. With the achievement of this objective we will be able to develop a rapid test that can be performed together with the retina photography. Potentially the protein rapid test can be performed at the diabetes patient's home using a kit provided by the family practitioner or the screening center via mail.

## 4. Conclusion

Our findings suggest that the maximal performance of this method has not been reached yet. Results can be improved in three potential ways: (i) by optimizing the parameter settings of both tear fluid proteomics and MA detector based classifiers; (ii) by comparing and choosing the best classifier available for the combined screening method; (iii) by fine tuning the patient examination and lab diagnostic protocols.

Considering that we intend to develop a prescreening method, in order to decrease the workload of the DR screening centers, with the maximization of our system sensitivity, in a subsequent study, the potential workload reduction also could be assessed.

Both tear sampling and retina photography are noninvasive methods and can be implemented at general practitioner's settings. For the assessment of cost-effectiveness of the method further analysis is needed. However, it is expected in the future that the cost of human resources in clinical care becomes higher, in parallel with a rapid decrease in the cost of IT and laboratory technologies. In light of these changes the combined method of tear fluid proteomics and computer-assisted image processing of digital retinal photographs may provide a promising alternative in DR screening.

## Figures and Tables

**Table 1:** Characteristics of the participants.

Total number of participants enrolled			Eye examination		
52			104		

Non-DR	DR		Eyes included	Eyes excluded	
	Proliferative	Nonproliferative		Tear fluid sampling	Retina photography

13	15	24	74	9	21

**Table 2:** Performance measures of the screening methods.

Screening method		SENS	SPC	ACC	PREC	NPV	*F*1	LRP	LRN
Image processing	Mean	0.84	0.81	0.84	0.94	0.63	0.89	4.42	0.20
	SD	0.11	0.04	0.10	0.13	0.11	0.11	2.36	0.11

Proteomics	Mean	0.87	0.68	0.82	0.89	0.63	0.88	2.72	0.19
	SD	0.17	0.12	0.11	0.21	0.15	0.16	1.24	0.12

Combined method	Mean	0.93	0.78	0.89	0.93	0.78	0.93	4.23	0.09
	SD	0.18	0.19	0.15	0.23	0.22	0.18	1.32	0.07

Performance measures of the image processing based, the tear proteomics based, and the combined screening methods. SENS: sensitivity, SPC: specificity, ACC: accuracy, PREC: precision (positive predictive value), NPV: negative predictive value, *F*1: *F*-measure, LRP: likelihood ratio positive, and LRN: likelihood ratio negative.
